# Complexes of Ruthenium(II) as Promising Dual-Active Agents against Cancer and Viral Infections

**DOI:** 10.3390/ph16121729

**Published:** 2023-12-15

**Authors:** Assunta D’Amato, Annaluisa Mariconda, Domenico Iacopetta, Jessica Ceramella, Alessia Catalano, Maria Stefania Sinicropi, Pasquale Longo

**Affiliations:** 1 Department of Chemistry and Biology, University of Salerno, Via Giovanni Paolo II, 132, 84084 Fisciano, Italy; asdamato@unisa.it (A.D.); plongo@unisa.it (P.L.); 2 Department of Science, University of Basilicata, 85100 Potenza, Italy; annaluisa.mariconda@unibas.it; 3 Department of Pharmacy, Health and Nutritional Sciences, University of Calabria, 87036 Arcavacata di Rende, Italy; domenico.iacopetta@unical.it (D.I.); jessica.ceramella@unical.it (J.C.); s.sinicropi@unical.it (M.S.S.); 4 Department of Pharmacy-Drug Sciences, University of Bari “Aldo Moro”, 70126 Bari, Italy

**Keywords:** ruthenium(II) complexes, dual antitumor/antiviral agents, *p*-cymene, triphenylphosphine, polypyridyl, *N*-heterocyclic carbenes

## Abstract

Poor responses to medical care and the failure of pharmacological treatment for many high-frequency diseases, such as cancer and viral infections, have been widely documented. In this context, numerous metal-based substances, including cisplatin, auranofin, various gold metallodrugs, and ruthenium complexes, are under study as possible anticancer and antiviral agents. The two Ru(III) and Ru(II) complexes, namely, BOLD-100 and RAPTA-C, are presently being studied in a clinical trial and preclinical studies evaluation, respectively, as anticancer agents. Interestingly, BOLD-100 has also recently demonstrated antiviral activity against SARS-CoV-2, which is the virus responsible for the COVID-19 pandemic. Over the last years, much effort has been dedicated to discovering new dual anticancer–antiviral agents. Ru-based complexes could be very suitable in this respect. Thus, this review focuses on the most recent studies regarding newly synthesized Ru(II) complexes for use as anticancer and/or antiviral agents.

## 1. Introduction

For many years, numerous researchers have actively worked in the field of inorganic drugs developing several metal complexes with diverse biological activities [1], such as anticancer [2,3,4,5,6,7,8] antibacterial [9], antioxidant [10], and antiviral [11,12,13]. During the COVID-19 pandemic [14], numerous studies have addressed using metal complexes in the hope of finding new strategies to cure the disease [15,16,17]. A comprehensive survey of the anti-COVID-19 options available using metal complexes has been recently reported by Gopal et al. (2023) [18]. Among the precious metals, ruthenium (Ru) has singular physicochemical properties, which makes it particularly useful in drug design [19]. Ru complexes represent an important class of metallo-organic compounds with numerous applications, and they are currently used in the fields of catalysis [20,21,22,23], including homogeneous, heterogeneous, and photocatalysis [24]. Moreover, numerous biological activities, such as antifungal [25], antibacterial [26], and anticarcinogenic [27,28,29,30,31,32], have been described for the complexes of Ru, as well as their uses in neurodegenerative diseases [33]. Several complexes with Ru(II) have been reported, including those with benzoic acid and their analogues [34], naphthoquinones, flavonoids, curcumins [35], *N*-heterocyclic carbenes (NHCs) [36], polypyridyl [37], phenanthroline [38], thiazole [39], Schiff bases [40,41,42,43], and half-sandwiched arene complexes [44]. Specifically, Ru complexes are widely studied in colorectal cancer [45], breast cancer [46], lung cancer [47], and prostate cancer [48]. Thota et al. (2018) recently described the importance of Ru(II) complexes as anticancer agents [49]. Ru(II) complexes show several advantages over traditional platinum-based chemotherapeutics, such as stability in biological media due to their higher redox potentials, which allows for longer circulation times in the body, thereby increasing the amount of time that the complexes have to target tumor cells [50]; selectivity towards tumor cells and minimal side effects, which are probably due to differences in the redox potentials or metal ion binding properties of tumor cells versus healthy cells [51]; easier accessibility for synthetic routes; low costs associated with the overall process; and, finally, Ru(II) complexes can be administered through a variety of routes, including oral, intravenous, and intraperitoneal. It is strongly believed that Ru(III) species act as prodrugs, and they are converted into Ru(II) species due to the hypoxic environment within the cancer cells [52,53,54]. Ru complexes are also studied in photodynamic therapy, photochemotherapy, and photothermal therapy [55]. With these activities, Ru can help to trigger antitumor activity only in desirable areas of the body or in cancer cells, apart from classical chemotherapeutic action [56,57]. Over the last two decades, the complexes of ruthenium have been also studied for their antioxidant [58], antimicrobial [59], and antiviral activities [60,61]. Moreover, the modulation activity of amyloid-β aggregation has been described, which can be useful in the treatment of Alzheimer’s disease [62,63]. Ru(II) and Ru(III) complexes are currently objects of great attention in the field of medicinal chemistry as antitumor agents with selective antimetastatic properties and low systemic toxicity [64,65,66,67]. The pharmacological activity of metal complexes can be attributed to either the metal itself, its ligands, or both, depending on the structure of the complex. The ruthenate anion itself may interact with cellular targets or simply act as a scaffold to carry bioactive ligands to a target site [26,68]. Ru-based compounds, as well as other metal complexes, act via a myriad of mechanisms, which usually involve interactions with DNA or various proteins such as enzymes and transcription factors [68]. Ru complexes, as well as platinum complexes, are generally defined as “multitargeted”, since they not only target DNA, but also contain either a vector to enable them to target cancer cells selectively and/or moieties that target enzymes, peptides, and intracellular proteins [69]. Several studies are addressed here to understand the mechanism of action of Ru(II) complexes. Recently, a probable mechanism of transfer hydrogenation catalysis with respect to anticancer activity has been described for Ru–arene complexes [70]. Moreover, a recent review on Ru(II) complexes suggested that metal-based candidate drugs are promising modulators of cytoskeletal and cytoskeleton-associated proteins [71]. Recently, Ru and rhodium complexes have been suggested as promising agents for metalloimmunotherapy [72].

**Figure 1:**
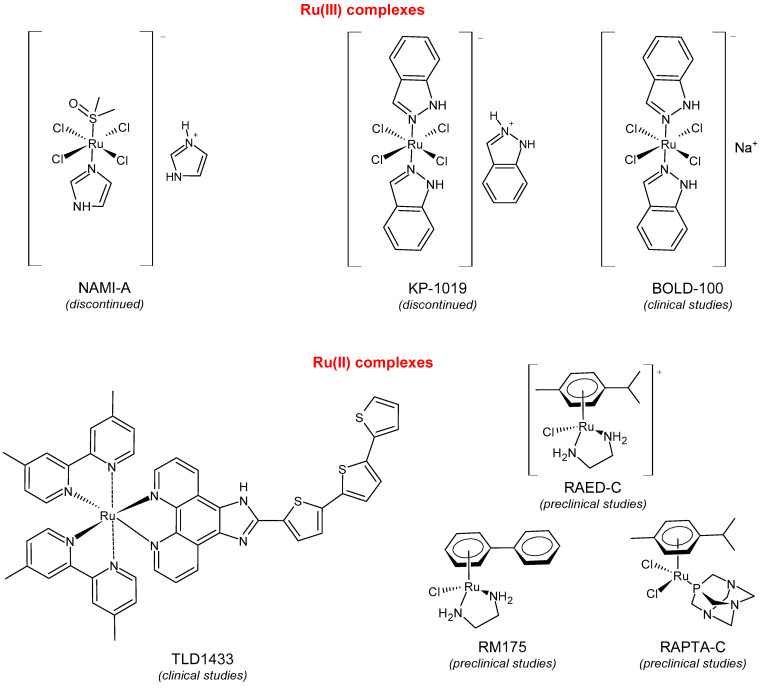
Structures of Ru(III) and Ru(II) complexes in clinical and preclinical trials.

In the fight against cancer, three Ru(III) coordination complexes (NAMI-A, KP1019, and BOLD-100) and one Ru(II) coordination complex (TLD1433) have advanced to clinical trials (Figure 1) [73]. Inside the tumor, Ru(III) is proposed to be activated by its reduction to Ru(II) due to prevalent reductive conditions. The Ru(III) complexes are tetrachloride complexes with axial N-heterocyclic ligands. NAMI-A exhibited strong inhibitory effectiveness against tumor malignancy and metastasis, thereby preventing the development of the growth of tumors. It entered phase II trials, but due to limited efficacy and acute side effects in many patients, it could not proceed further for clinical development [74]. The Ru(III) complex sodium BOLD-100 is among the most widely investigated nonplatinum metal-based anticancer drugs [75]. It was studied as a substitution of the Ru complex KP1019, which entered phase I trials for colorectal tumors, but its further development was halted due to its low solubility [76]. KP1019 is known to be active against primary tumors, while NAMI-A is active against secondary tumors *via* antiangiogenic and antimetastatic activities [6]. NAMI-A and KP1019 have been shown to bind to DNA, RNA, and proteins [77]. The octahedral polypyridyl Ru(II) complex TLD1433 has potential as a photosensitizer for photodynamic therapy in the treatment of bladder cancer [78]. 

Ru(II) complexes, namely RM175, RAED-C, and RAPTA-C, are 18-electron Ru–arene “piano-stool” complexes, in which an η^6^-arene ring stabilizes the 2^+^ oxidation state of the Ru metal center [73]. These complexes entered into preclinical studies because of their appealing anticancer properties [79]. RM175 was the first Ru(II) complex reported to have potential for anticancer activity. RM175 has undergone successful in vitro and in vivo cytotoxic assessment and has shown efficient cytotoxicity in vitro, with IC_50_ values similar to that of cisplatin [80]. RM175 shows a mechanism of action similar to cisplatin through its interaction with guanine. The possible mechanism of interaction has been recently elucidated by Prathima et al. (2023) [6]. However, it differs from cisplatin, as it revealed no cross-resistance against cisplatin-resistant ovarian carcinoma cells (A2780cis); this is indicative of a distinctive mode of anticancer action and has also been reported to trigger p53-dependent cell-cycle arrest [81]. Ru(II)–arene RAED-type compounds (ED = ethylenediamine) and Ru(II)–arene RAPTA-type compounds (PTA = 1,3,5-triaza-7-phosphaadamantane or 1,3,5-triaza-7-phosphatricyclo-[3.3.1.1]decanephosphine) were developed by the groups of Sadler [82] and Dyson [83], respectively. Both have the *p*-cymene moiety, that is, 1-methyl-4-(propan-2-yl)benzene. The RAED series was first reported in 2001 by Morris et al. [84], and these compounds are able to coordinate with DNA through the N7 of guanine residues and, when bearing an extended arene ligand such as biphenyl, dihydroanthracene, or tetrahydroanthracene, may concomitantly intercalate in DNA. These compounds are cytotoxic against diverse cancer cell lines, including cisplatin-resistant strains [85]. Swaminatan et al. (2022) [86] reported that RAED-C is highly active in primary tumors, whereas RAPTA-C is inactive in primary tumors but possesses antimetastatic and antiangiogenic properties. Moreover, the former preferentially forms adducts at the DNA sites with only one additional binding site at the histone level, while the latter preferably forms adducts at the histone protein sites residing on the surface of the nucleosome core. Hildebrandt et al. (2022) [87] have recently reported that both compounds, RAPTA-C and RM175, are being studied in advanced clinical studies. However, to our knowledge, no other research confirms this statement. 

Moreover, the drug delivery forms of Ru complexes have also been studied as antitumor drugs for combination therapy [88]. Finally, and very importantly, dual-active drugs are a concept that has been noted as an imperative in future drug design. The development of novel drugs that can have double biological behavior (anticancer–antiviral, anticancer–antimicrobial, etc.), leading to the opportunity to treat two different diseases, has been recently widely addressed [89,90,91,92]. In this context, this review focuses on the Ru complexes in clinical trials and on the most promising drugs in preclinical studies as antitumoral and antiviral agents, thereby highlighting their importance in the treatment of different types of cancer and their potential as antiviral drugs. We used Scopus, PubMed, Google Scholar, MEDLINE, and ScienceDirect to review the literature on Ru(II) complexes over the last four years. The search criteria considered the occurrence of the combination of the following keywords: “ruthenium(II)”, “Ru(II)”, “anticancer”, “antitumor”, “antiviral”, and “COVID-19”, which were found either in the title and abstract or in the text.

## 2. Ruthenium(II/III) Complexes in Clinic Trials and Advanced Preclinical Studies as Anticancer Agents

### 2.1. BOLD-100

The Ru(III) complex sodium *trans*-tetrachlorobis(1*H*-indazole)ruthenate(III) (BOLD-100, formerly known as NKP-1339, KP1339, and IT-139) is a double prodrug that undergoes hydrolysis via the ligand exchange of chloride ligands and subsequent reduction to Ru(II) [93,94]. BOLD-100 is a versatile small molecule with manifold intracellular modes of action, which were previously summarized by the research group that synthesized this molecule [95]. In clinical phase I evaluation, BOLD-100 therapy led to disease stabilization and even partial response in various types of advanced solid tumors, including colorectal cancer, non-small-cell lung cancer, and neuroendocrine tumors of carcinoid origin [96]. BOLD-100 was granted an orphan drug designation (ODD) in gastric and pancreatic cancers [97]. It is currently in a phase 2a clinical trial in combination with folinic acid, 5-fluorouracil, and oxaliplatin (FOLFOX regimen) for the treatment of advanced solid tumors, such as colorectal, pancreatic, and gastric cancers, as well as cholangiocarcinoma (NCT04421820) [98,99]. Moreover, BOLD-100 has also demonstrated increased activity in the cell lines from esophageal cancer, blood cancers, and bladder cancer [100]. BOLD-100 has also recently gained particular interest for its potential multiple activities. Earlier, the drug had won orphan drug titles for its indication of pancreatic cancer [98,100]. Besides its undiscussed anticancer activity, it has been recently demonstrated that this compound is also a potent inhibitor of the replication of human immunodeficiency virus type 1 (HIV-1), human adenovirus type 5, and SARS-CoV-2 in vitro [101]. Repression of the genes involved in DNA repair, the induction of reactive oxygen species (ROS), and interference with ribosomal proteins seem to be results of BOLD-100 activity [75]. Moreover, BOLD-100 is an inhibitor of glucose-regulated protein 78 kDa (GRP78) (WO/2017/151762), thus disrupting endoplasmic reticulum homeostasis, inducing endoplasmic reticulum stress, and eliciting an unfolded protein response [102]. This is reflected by the phosphorylation of the eukaryotic translation initiation factor 2A [103] and caspase-8-dependent cell death [104]. The suppression of Grp78 transcription is a mechanism described for antiviral activity, which has also been demonstrated against SARS-CoV-2 [105]. Moreover, in vitro studies have demonstrated that this compound triggers an immunogenic cell death (ICD) signature hallmarked by the phosphorylation of PERK, the eukaryotic translation initiation factor 2α (eIF2α) exposure of calreticulin on the cell membrane, the release of the high mobility group box 1, and the secretion of ATP [106]. Interestingly, Mucke (2022) [107] reported that BOLD-100 inhibited the cytopathic activity in an assay based on Vero-E6 cell lines infected with the Wuhan strain of the virus: the absolute EC_50_ value for preinfection protection by BOLD-100 was 1.9 μM, whereas postinfection treatment required 1.8 μM. This value is orders of magnitude lower than the 200–400 mM cytotoxicity limit for BOLD-100 in this cell line, and it is much lower than the respective values for the antiviral remdesivir [108]. At 200 μM, the cytopathy of 293T-ACE2 human kidney cells (which express the ACE2 receptor) infected with the ‘California variant’ of the B.1.1.7 viral strain was prevented by BOLD-100 [107]. Yet, a general limitation of systemic cancer therapy efficacy is the acquisition of treatment resistance [109]. The mechanism against solid tumors that has been recently suggested is related to its ability to inhibit glycolysis and render cells vulnerable to glucose-deficient metabolism [110]. It is known that, besides other metabolic changes, including alterations in oxidative phosphorylation or glutaminolysis [111], several types of solid cancers show improved glycolysis to convert glucose to lactate, even under aerobic conditions: this effect is called the “Warburg effect” [112]. BOLD-100 demonstrated a significant glycolysis-blocking anti-Warburg effect as a novel mechanism of action. Thus, glycolysis inhibition has also been suggested as a potential strategy to overcome acquired BOLD-100 resistance and enhance BOLD-100 anticancer activity. Moreover, an upregulated glucose uptake was detected in combination with BOLD-100 exposure [110]. Baier et al. (2023) [113] recently identified BOLD-100 as an epigenetically active substance targeting several oncometabolic pathways. The authors suggested that acquired BOLD-100-resistant colon and pancreatic carcinoma cells may be related to lipid metabolism. BOLD-100 significantly reduced the production and release of lactate, which is a major immunosuppressive metabolite. The existence of crosstalk between BOLD-100 exposure, acquired resistance, and histone acetylation has been suggested.

### 2.2. TLD1433

TLD1433 (also known as Ruvidar^®^ and “Theralase^®^) was the first Ru(II)-based photosensitizer to enter clinical trials and successfully complete a phase 1b human clinical trial (NCT03053635). A phase 2 study is ongoing (NCT03945162) [114,115] to evaluate TLD1433 in non-muscle-invasive bladder cancer patients. It has been recently suggested as a repositioning drug for the treatment of conjunctival melanoma, which is a rare but often deadly ocular cancer [116], and human lung adenocarcinoma [117]. Recently, Karges (2022) [118] reviewed the clinical development of TLD1433 and other metal-containing compounds, including rostaporfin (Purlytin^®^), motexafin lutetium (Lutrin^®^/Antrin^®)^, and the sulfonated aluminium phthalocyanin (Photosens^®^), bearing the different metals Sn, Lu, and Al, respectively, as well as padeliporfin (WST09) and padeliporfin (WST11 or TOOKAD^®^ soluble), which contain Pd, as photosensitizers for the photodynamic therapy of cancer.

### 2.3. RAPTA-C

The therapeutic potential of Ru(II)–arene RAPTA-type compounds (PTA = 1,3,5-triaza-7-phosphaadamantane or 1,3,5-triaza-7-phosphatricyclo-[3.3.1.1]decanephosphine) has been thoroughly investigated, thus owing to the excellent antimetastatic property of the initial candidate RAPTA-C [Ru(η^6^-*p*-cymene)Cl_2_(PTA)] [119]. It is a multitargeting drug candidate that has demonstrated pH-dependent DNA damage, inhibited the enzyme activity of cathepsin-B and thioredoxin reductase, and showed selectivity towards the hypoxic environment of cancer cells [120]. It represents an innovative antitumor therapy and a better-tolerated alternative to Pt-based chemotherapeutic drugs in the treatment of tumors, as it exhibits antitumoral, antimetastatic, and antiangiogenic activities through protein and histone–deoxyribonucleic acid alterations [121]. RAPTA-C acts synergistically in association with other drugs, such as the EGFR inhibitor erlotinib, the tyrosine kinase inhibitor axitinib, PI3K, and the mTOR inhibitor BEZ-235, as demonstrated by in vivo models [122,123,124,125]. The study by Weiss et al. (2014) [126] demonstrated that RAPTA-C caused a reduction in the growth of primary tumors in preclinical models for ovarian (A2780 ovarian carcinoma transplanted onto a chicken chorioallantoic membrane model) and colorectal (in LS174T colorectal carcinoma in athymic mice) carcinomas. Moreover, the clearance rate of RAPTA-C from the organs and the bloodstream was studied using RAPTA-C that incorporated radio-labeled (^103^Ru). Biodistribution studies with radio-labeled (^103^Ru) RAPTA-C demonstrated that the compound is rapidly cleared from the organs and the bloodstream through excretion by the kidneys. Recently, the combination of RAPTA-C and paclitaxel based on fructose-coated nanoparticles has been suggested as a dual drug delivery system for the treatment of metastatic cancer. The dual drug delivery system was studied via in vitro tests using MDA-MB-231 breast cancer cells, and it was observed that RAPTA-C, in combination with paclitaxel, significantly enhanced antitumor and antimetastatic action [127].

## 3. Ruthenium Complexes Acting against Viruses

Several metal-based drugs have been described regarding their antiviral activities, thereby highlighting the potential for these metal-based drugs to be used in treating COVID-19 [17,128,129,130,131]. Although many studies have described the anticancer activity of Ru complexes, there are very few reports on their antiviral activity [129,132,133]. Recently, Gil-Moles and colleagues (2021) [134] described some metallodrugs, including Ru complexes, and their activity against SARS-CoV-2. Some complexes were potent inhibitors of essential SARS-CoV-2 targets, such as the SARS-CoV-2 spike protein/host ACE2 receptor interaction and the SARS-CoV-2 papain-like protease (PL^pro^). Moreover, Janković et al. (2022) [135] reported other Ru complexes as potent antivirals against SARS-CoV-2, which target the papain-like proteases PL^pro^ and M^pro^. They are shown in the next paragraphs. De Oliveira et al. (2020) [61] described their antiviral activity against other viruses, such as the Chikungunya virus, thereby highlighting the potential of Ru-based compounds as broad-acting antivirals.

## 4. Preclinical Studies on Ru(II) Complexes

### 4.1. Preclinical In Vitro and In Vivo Studies on Ru(II) Complexes as Anticancer Agents

Recent studies regarding the antitumor activities of Ru(II) complexes have been reported in Table 1. The IC_50_ values (the concentration that kills or inhibits the cell viability by 50%) reported in the table were obtained from a colorimetric assay (MTT) and via a water-soluble tetrazolium salt (WST-1) assay against different cell lines. In one article, a growth inhibition of 50% (GI_50_) was reported, using the MTT (3-[4,5-dimethylthiazole-2-yl]-2,5-diphenyltetrazolium bromide) assay. 

Shereef et al. (2022) [136] studied the cytotoxic activity of the complex [Ru(NO)(Et_2_NpyS_4_)]Br (**1**) and its ligand against human hepatocellular carcinoma (HepG2) cell lines and normal (BNL) cell lines at different concentrations using a WST-1 assay. The IC_50_ values of the cancer cells were lower than those of the normal cells, thereby indicating that both compounds may be selective and effective towards cancer cells. The in vitro protein binding to bovine serum albumin (BSA) was also studied, and a mechanism was proposed. The Ru center improved the reaction rate through coordination affinity and changed the binding process. A molecular docking study also supported the obtained results, thus showing that Ru complex **1** is located in the IA pocket (Trp134) with a binding affinity (−7.27 kcal/mol) that is slightly lower than the ligand (−8.05 kcal/mol), and the results were in agreement with the binding constants. 

Gurgul et al. (2022) [137] studied the involvement of three polypyridyl Ru(II) complexes (**2**–**4**) in the formation of metastases and the regulation of cell adhesion properties. In vitro antitumor activity was evaluated against A375 and A2058 melanoma cell lines, against MCF-7 and MDA-MB-231 breast cancer cell lines, and against the noncancerous immortalized keratinocyte HaCat. The IC_50_ values are reported in Table 1 and compared to cisplatin used against the cell lines mentioned above (IC_50_ = 61 ± 5 µM; 53 ± 9 µM; 54 ± 6 µM; and 82 ± 3 µM, respectively, against cancerous cells). Moreover, the cytotoxicity against the HaCat cells was lower than that against the cancer cells for all three Ru complexes. The three complexes impacted the activity of the selected integrins and upregulated the expression of focal adhesion components such as vinculin and paxillin, thereby leading to an increased number of focal adhesion contacts. All three complexes interfered with crucial metastasis processes: they markedly decreased migration, invasion, and transmigration at much lower doses than the cytotoxic dose. The most significant changes in cell adhesion and motility were observed with complex **4**, which was also the most cytotoxic against MDA-MB-231 cells. 

Cseh et al. (2022) [138] described the synthesis and cytotoxic activity evaluation of Ru(II) complexes with phthiocol against CH1/PA-1 teratocarcinoma cells, SW480 colon carcinoma cells, and A549 non-small-cell lung cancer cells using an MTT assay (after 96 h). The standard drugs used were cisplatin (IC_50_ = 3.8 ± 1.0 µM; 2.3 ± 0.2 µM; and 0.073 ± 0.001 µM, respectively), carboplatin (IC_50_ = 38 ± 3 µM; 42 ± 10 µM; and 0.79 ± 0.11 µM, respectively), and oxaliplatin (IC_50_ = 0.98 ± 0.21 µM; 0.29 ± 0.05 µM; and 0.18 ± 0.01 µM, respectively). Complexes **5** and **6** with a *p*-cymene and biphenyl arene, respectively, were the most promising compounds. The possible correlation between the cytotoxicity, cellular accumulation, and lipophilicity was evaluated by quantifying the total cellular Ru using ICP-MS: the most cytotoxic compounds, **5** and **6** (with the highest aqueous stability), yielded the highest total ruthenium content in the cell lysates. Complex **7** showed higher cellular accumulation, with a magnitude comparable to that of complexes **5** and **6**, even though it showed lower stability in the aqueous medium. A positive correlation was found between the cytotoxicity, lipophilicity [139], and cellular accumulation of the compounds: complexes with higher calculated miLogP values for the arene showed significantly higher cellular Ru levels. Cell-cycle studies evidenced that the compounds had a stronger impact on the SW480 cells than on the CH1/PA-1 cells. Data from the apoptosis assay revealed a pronounced increase in early and late apoptotic cells by complexes **5** and **6** in the SW480 cells.

Juszczak et al. (2022) [140] described the synthesis of four Ru(II) complexes and evaluated their cytotoxicity effects against leukemic HL-60 cells and normal peripheral blood mononuclear cells (PBMCs). The complex η^5^-cyclopentadienyl)Ru(CO)_2_(*η*^1^-*N*-maleimidato (**8**) showed high cytotoxicity and genotoxicity against both cell types, but it was 10 times more cytotoxic against HL-60 cells compared to PBMCs, whereas complexes (*η*^5^-cyclopentadienyl)Ru(CO)_2_-*N*-ethoxysuccinimidato (**9**) and *η*^5^-cyclopentadienyl)Ru(CO)_2_-*N*-phthalimidato (**10**) were only cytotoxic against cancerous cells at the highest concentrations used. The succinimide complex **9** enhanced the viability of the PBMCs. The maleimido complex **8** was the most interesting compound of the series: it arrested the cell cycle in the sub-G1 phase and induced apoptosis. 

Liang et al. (2022) [141] described the synthesis of three polypyridyl Ru(II) complexes (**11**–**13**; IPP = 4-(1*H*-imidazo[4,5-f][1,10]phenanthrolin-2-yl)-*N*,*N*-diphenylaniline) and investigated their anticancer efficacy in vitro and in vivo. The in vitro assays were carried out on cancerous cell lines, specifically B16 (mouse melanoma), HepG2 (human hepatocellular carcinoma), and A549 (human lung) cells, as well as normal LO2 (human normal embryonic liver) cells. The complexes showed higher cytotoxic activity against B16 cell lines than against HepG2 and A549 cell lines in comparison to cisplatin (IC_50_ = 20.5 ± 0.8 µM; 11.4 ± 0.8 µM; and 11.1 ± 0.7 µM, respectively, against cancerous cells). All complexes showed lower cytotoxicity than cisplatin against normal cells. Therefore, the B16 cell line was selected for subsequent studies. Cytotoxicity, scratching, and colony-forming studies demonstrated that complexes **11–13** could effectively inhibit the cell proliferation and migration ability of the cells. Mitochondrial localization, membrane-potential studies, and the detection of reactive oxygen species showed that these complexes directly accumulate in the mitochondria; then, the complexes cause a decline in the mitochondrial membrane potential and induce an increase in the intracellular reactive oxygen species (ROS) levels. It was found that the complexes inhibited the growth of B16 cell lines at the G0/G1 phase through cell-cycle studies. Moreover, it was demonstrated that the complexes can cause early apoptosis in B16 cell lines and could regulate the expression of Bcl-2-family proteins. Then, antitumor activity in vivo experiments, carried out on a B16 black mouse xenograft tumor model, demonstrated that complex **12** (10 mg/kg) could effectively inhibit tumor growth with a high inhibitory rate (65.95%).

Cervinka et al. (2022) [142] investigated the antitumoral activity of complexes containing a tridentate tris(1-pyrazolyl)methane ligand against a panel of human cancer cell lines (MCF-7 for breast cancer; HeLa for cervical cancer; 518A2 for melanoma; HCT116 for colon cancer; and RD for rhabdomyosarcoma) and against normal human fibroblasts, MRC5pd30 cells, to assess the toxicity of the complexes. Complexes **14**–**16** were the most interesting, which were active and generally selective, as well as showed higher or similar activity to cisplatin against cancerous cells (IC_50_ = 13 ± 3 µM; 14 ± 3 µM; 2.6 ± 0.7 µM, 8 ± 1 µM; and 4.6 ± 0.3 µM, respectively). The cytotoxic effects of **14**–**16** on noncancerous MRC5pd30 cells were significantly lower, thereby demonstrating selectivity toward cancer cells over noncancerous cells. The authors also demonstrated that these complexes inhibited cancer cell growth by disrupting mitochondrial calcium homeostasis.

Priya et al. (2023) [143] recently studied two mononuclear Ru(II) polypyridyl complexes (**17** and **18**) for their antitumoral and antimicrobial activities. Complex **18** showed higher antiproliferative activity than **17** against HeLa cervical cancer cell lines, which was measured via MTT assay. However, no cytotoxicity study was reported on healthy cell lines, and IC_50_ values were reported for standard drugs for comparison. 

Křikavová et al. (2023) [144] described two metal complexes that each have thiadiazole moiety. Specifically, the Ru complex **19** [Ru(η^6^-pcym)(L1)Cl]PF_6_ presents a *p*-cymene moiety. In vitro antitumor activity was determined against cisplatin-sensitive (A2780) and -resistant (A2780cis) ovarian cancer cell lines and healthy cell lines (CCD-18Co for the colon and CCD-1072Sk for foreskin fibroblasts). Complex **19** exhibited moderate inhibitory effects on the metabolic and proliferation activities of the cancer cells tested compared to cisplatin (IC_50_ = 3.29 ± 0.88 µM and IC_50_ = 11.96 ± 2.71 µM, respectively, against cancerous cells). However, it showed an inhibitory effect against CCD-1072Sk healthy cells (IC_50_ = 3.29 ± 0.88 µM after 48 h compared to the 26.39 ± 8.22 µM value of cisplatin).

Recently, de Araujo-Neto et al. (2023) [145] presented the in vitro and in vivo studies of half-sandwich Ru complexes (**20**–**22**) with alizarin, specifically Ru/arene/alizarin, as antitumor agents. The cell lines used in their in vitro assays were MDA-MB-231 and MCF-7 breast cancer cell lines and A549 lung tumor cell lines against the nontumor cell lines MCF-10A and MRC-5, respectively. Cisplatin was used as a standard (IC_50_ = 10.2 ± 0.2 μM; 8.6 ± 1.8 μM; and 14.4 ± 1.4 μM, respectively, against cancerous cell lines). Complexes **20** and **21** were more selective against the two breast tumor cell lines, with **21** being the most cytotoxic toward MDA-MB-231 cell lines in yielding an IC_50_ value comparable to that of cisplatin (IC_50_ = 6.5 μM). Complex **20** exhibited strong covalent DNA interaction, whereas it was weak for **21**. Complexes **20** and **21** inhibited colony formation and induced cell-cycle arrest in the sub-G phase in MDA-MB-231 cell lines in a concentration-dependent manner. Complex **21** inhibited colony formation and had a potential antimetastatic action, thereby impeding cell migration in the wound-healing experiment. In vivo, toxicological experiments showed that **20** and **22** demonstrated the most zebrafish embryo developmental toxicity (inhibition of spontaneous movements and heartbeats), whereas **21** revealed the lowest toxicity; thus, complex **21**, bearing the triphenylphosphino moiety, was suggested as the most promising candidate for drug development to treat triple-negative breast cancer.

Bresciani et al. (2023) [146] presented a study regarding the antitumor potential of several dinuclear Ru biscyclopentadienyl carbonyl complexes (**23**–**26**) against A549 (lung), SW480 (colon), and A2780 and A2780cis (ovarian) cancer cell lines, as well as the nontumoral HEK-293 cell line. Cisplatin was used as the standard (IC_50_ = 43 ± 3 µM, 35 ± 2 µM, 8.3 ± 1.4 µM, and 30 ± 3 µM, respectively, against cancer cell lines). Complexes **24** and **26** were mixtures of stereoisomers (**24a** and **24b** and **26a** and **26b**), whereas complexes **23** and **25** occurred as one single isomer. Specifically, complex **6** was an aspirin derivative. Complexes **24**–**26** showed cytotoxic activities that were similar to the reference in the A549, SW480, and A2780 cancer cells, whereas all the complexes, including **23**, overcame cisplatin resistance in the A2780cis cells. Moreover, complexes **23**, **24**, and **26** increased the intracellular ROS levels, which were likely responsible for the antitumor action. The mechanism of action of the complexes could also be related to binding with DNA or RNA and possibly ascribable, at least in part, to the derivatives formed via the modification of the hydrocarbyl ligand. Moreover, the authors suggest albumin protein as a possible vehicle for the transportation and delivery of the complexes through the establishment of hydrophobic interactions.

A successive study by Bresciani et al. (2023) [147] presented the synthesis and antiproliferative activity of several Ru(II) complexes against nine human cancer cell lines (human ovarian carcinoma, A2780; cisplatin-resistant human ovarian carcinoma, A2780cisR; breast adenocarcinoma, MCF-7; human osteosarcoma, HOS; human lung adenocarcinoma, A549; human pancreatic carcinoma, PANC-1; human colorectal adenocarcinoma, Caco-2; human prostate carcinoma, PC-3; and human cervical carcinoma, HeLa) and normal human lung fibroblast (MRC-5) cells. Complexes **27** and **28**–**30** showed higher cytotoxicity effects than cisplatin, with **29** being the most active. Cisplatin was used as the standard (IC_50_ = 15.2 ± 1.1 µM; 40.0 ± 3.9 µM; 28.4 ± 2.7 µM; 26.3 ± 3.3 µM; 39.2 ± 3.1 µM; >50 µM; >50 µM; >50 µM; and 30.7 ± 0.6 µM, respectively, against cancerous cells); the IC_50_ value for RAPTA-C was >50 µM against all the cell lines. Moreover, inductively coupled plasma mass spectrometry cellular uptake studies were carried out in the A2780 cells, thereby showing a higher level of internalization for **29** and **30** compared to **27**, **28**, and RAPTA-C. An interesting impact of **28** and **29** was noted in the cell cycle, thereby leading to the majority of the cells being arrested in the G0/G1 phase. Furthermore, **28** moderately induced apoptosis and oxidative stress, while **29** triggered autophagy and mitochondrial membrane-potential depletion.

Nayek et al. (2023) [148] presented studies on the antitumor activities of Ru(II)–arene benzimidazole complexes (**31**–**33**) that bear *p*-cymene moiety. The antitumor activity was evaluated against HeLa and MCF7 cancer cell lines and HEK 293 normal cells. Cisplatin was used as the standard (IC_50_ = 16.20 ± 0.28 µM and 21.19 ± 0.66 µM, respectively, against cancer cell lines). Complex **32**, bearing a triphenylphosphine moiety, was the most active against both of the malignant cell lines. Complexes **31** and **33** also showed higher activity than cisplatin in the HeLa cells.

Schoeller et al. (2023) [149] reported on the synthesis and cytotoxic activity evaluation of bipyridine Ru(II) complexes with halogen-substituted salicylates against breast cancer (MCF-7) and glioma (U-118MG) cell lines using an MTT assay. Complex **34** was the most effective against the MCF-7 cell lines, whereas complexes **35**–**37** showed antiproliferative effects against the U-118MG cell lines. Complex **35** showed the lowest IC_50_ value after 24 h of incubation, and complex **37** showed the lowest IC_50_ value after 48 h of incubation. All the complexes could interact with BSA, with complex **37** being the one with the highest value with respect to its binding constant. The complexes appeared to be able to interact with DNA; they likely intercalated into the double-stranded DNA structure, as assessed by the ability of the complexes to displace ethidium bromide (EB) from the EB–DNA complex.

Alguacil et al. (2023) [150] recently presented a study of two tetranuclear complexes of Ru(II) coordinating CuCl_2_ and NiCl_2_ fragments (**38** and **39**, respectively) against six human solid tumors, namely, A549 (lung), HBL-100 (breast), HeLa (cervix), SW1573 (lung), and WiDr (colon). The two complexes each showed excellent antiproliferative activity, with nanomolar GI_50_ values (cisplatin was used as the standard drug: GI_50_ = 4933 ± 180 nM; 1866 ± 162 nM; 1787 ± 518 nM; 2746 ± 375 nM; 16,846 ± 3258 nM; and 22978 ± 4316 nM, respectively). The subsequent transformation of complexes **38** and **39** in the respective heterobimetallic complexes was demonstrated. The mechanism of action of these compounds was also deepened by using a colony-formation assay for the SW1573 cells and cell-death-mechanism assay for the HeLa cells. In the former, the presence of the two complexes led to a reduction in the size and density of the colonies; in the latter, both of the complexes induced apoptosis, with complex **39** doing so faster than complex **38**. The interaction of the complexes with a pBR322 DNA plasmid was also evaluated: neither **38** nor **39** modified the mobility of the plasmid. The authors suggested a different action mechanism from that of cisplatin. However, no studies of the cytotoxicity were carried out on healthy cells. 

Mitchell et al. (2023) [151] have recently reported a study on triarylphosphine-coordinated bipyridyl Ru(II) complexes inducing mitochondrial dysfunction. Cytotoxicity assays were carried out against leukemic HL-60, lung A549, prostate adenocarcinoma DU145, and cervical HeLa cell lines. Complexes **40** and **41** were the most interesting of the study when compared to cisplatin (IC_50_ = 1.06 ± 0.15 µM (after 72 h) and 7.34 ± 0.82 µM (after 24 h) against HL-60 cells, and IC_50_ = 5.49 ± 1.30 µM; 1.44 ± 0.35 µM; and 3.98 ± 0.81 µM, against A549, DU145, and HeLa cell lines, respectively, after 24 h). The 4,4′-dimethylbipyridyl-substituted complex **41** showed strong depolarizing capabilities; this depolarization was selective for the mitochondrial membrane and occurred within minutes of treatment in the cancer cells. In the depolarized mitochondrial membranes, complex **41** showed an eight-fold increase, which was higher than the one observed by the carbonyl cyanide chlorophenylhydrazone (two-fold increase) that was used as a reference. Furthermore, the study revealed a strong binding affinity between the compound and DNA through an intercalative binding mode. This was confirmed by EB displacement and viscosity-measurement studies. 

Das et al. (2023) [152] studied two Ru(II) carbonyl complexes (**43** and **43**) and their X-ray structures, DNA/BSA protein binding, and antiproliferative activity against human breast cancer (MCF-7), human lung cancer (A549), triple-negative breast cancer (MDA-MB-231), and gastric adenocarcinoma (AGS) cell lines, as well as normal (WRL68) cells, using an MTT assay. The complexes were compared to cisplatin (IC_50_ = 14.2 ± 1.6 µM; 15.2 ± 2.8 µM; 90.8 ± 2.1 µM; and 27.1 ± 2.3 µM, respectively, against cancerous cells). Interestingly, both of the complexes showed higher activity effects than the reference against MCF-7 cell lines. Moreover, a good binding affinity with DNA was observed through an intercalative binding mode, which was further confirmed by EB displacement and viscosity-measurement studies.

Ceramella et al. (2023) [153] reported on the synthesis of six Ru(II)–NHC complexes and evaluated their biological activities, including anticancer, antimicrobial, and antioxidant. Cytotoxicity evaluation was studied against the breast cancer cell lines MDA-MB-231 and MCF-7; neuroblastoma cells SH-SY5Y; and the nontumoral cells MCF-10A and BALB/3T3. Complexes **44** and **45** were the most active compounds compared to cisplatin (IC_50_ = 32.15 ± 1.0 µM; 26.19 ± 1.1 µM; and 18.75 ± 0.9 µM, respectively, against cancerous cells). They showed inhibitory activity effects regarding the human topoisomerase I and triggered cell death by apoptosis. Moreover, they all possessed the best antibacterial activity effects against Gram-positive Staphylococcus aureus, at a concentration of 25 µg/mL, and a high ability with respect to inhibiting ABTS^•+^ in an ABTS assay compared to the well-known antioxidant Trolox.

Kavukcu et al. (2023) [154] described two Ru(II)–*p*-cymene complexes (**46** with an aliphatic chain group and **47** with N,S,S triple coordination), which were investigated regarding their antitumoral activity effects against HepG2 cell lines using an MTT assay and focusing on cell death mechanisms. Both complexes were more active than cisplatin. Complexes **46** and **47** reduced the cell viability to 50% at approximate concentrations of 10 µM against HepG2 cell lines. In normal Vero cells, **46** showed almost the same activity, whereas **47** was even more active than it was against tumor cells. The IC_50_ values were not given.

Chen et al. (2023) [155] recently reported an interesting study on two polypyridyl Ru(II) complexes (**48** and **49**) and their cytotoxic activities in vitro toward A549 (lung adenocarcinoma), HepG2 (human hepatocellular carcinoma), SGC-7901 (gastric adenocarcinoma), HeLa (cervical cancer), BEL-7402 (hepatocellular carcinoma), and B16 (mouse melanoma) cells, as well as noncancer LO2 (hepatic fibroblast) cells, which were investigated using the MTT method. Unexpectedly, complexes **48** and **49** did not prevent these cancer cells’ proliferation (IC_50_ > 200 µM, respectively, against all the cell lines). However, the liposomes entrapping the complexes (**48lipo** and **49lipo**) exhibited high anticancer efficacy effects, especially toward the SGC-7901 cell lines. Cisplatin was used for comparison (IC_50_ = 6.7 ± 0.4 µM; 9.3 ± 0.8 µM; 5.7 ± 0.2 µM; 5.8 ± 0.5 µM; 15.2 ± 1.4 µM; and 19.6 ± 2.2 µM, respectively). The cell-colony, wound-healing, and cell-cycle distribution demonstrated that the complexes inhibited the cell growth at the G2/M phase. Studies on the apoptosis showed that both **48lipo** and **49lipo** could effectively induce apoptosis via regulation of the Bcl-2-family proteins’ expression. They also improved the ROS and malondialdehyde levels, which inhibited the generation of glutathione and finally led to ferroptosis. In vivo experiments showed that **48lipo** could prevent tumor growth in a concentration-dependent manner with a high inhibitory rate (53.53% and 72.90% for 1.23 mg/kg and 2.46 mg/kg of **48lipo**, respectively). Furthermore, hematoxylin–eosin stain results showed that **48lipo** did not cause chronic organ damage toward the heart, liver, lung, spleen, kidney, and brain, and it strongly promoted the necrosis of solid tumors.

**Table 1:** In vitro and in vivo studies on Ru(II) complexes for use as anticancer agents.

Structure	Compound	Cytotoxicity Studies	Ref.
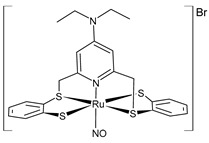	[Ru(NO)(Et_2_NpyS_4_)]Br **(1)**	IC_50_ = 53 ± 1.3 µg/mL (HepG2)	Shereef et al. 2022 [136]
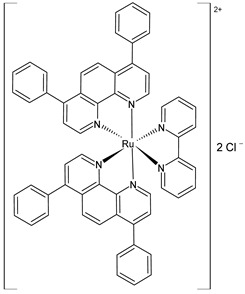	[Ru(dip)_2_(bpy)]Cl_2_, **(2)**	IC_50_ = 9.7 ± 0.4 µM (A375) IC_50_ = 4.9 ± 0.9 µM (A2058) IC_50_ = 3.9 ± 0.6 µM (MCF7) IC_50_ = 0.8 ± 0.6 µM (MDA-MB-231)	Gurgul et al. (2022) [137]
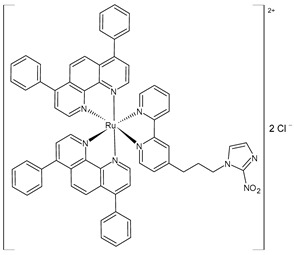	[Ru(dip)_2_(bpy-NitroIm)]Cl_2,_ **(3)**	IC_50_ = 11.2 ± 0.9 µM (A375) IC_50_ = 10.8 ± 0.8 µM (A2058) IC_50_ = 13 ± 2 µM (MCF7) IC_50_ = 3.8 ± 0.2 µM (MDA-MB-231)	Gurgul et al. (2022) [137]
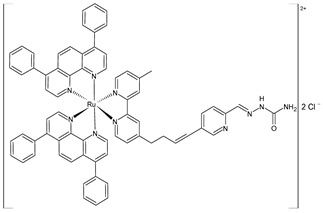	[Ru(dip)_2_(bpy-NitroIm)]Cl_2_ **(4)**	IC_50_ = 15.0 ± 0.6 µM (A375) IC_50_ = 4.7 ± 0.5 µM (A2058) IC_50_ = 13.1 ± 0.3 µM (MCF7) IC_50_ = 1.8 ± 0.3 µM (MDA-MB-231)	Gurgul et al. (2022) [137]
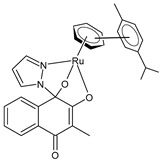	[3-Methyl-4-oxo-(1*H*-κN^2^-pyrazol-1-yl)-1,4-dihydronaphtalene-1,2-bis(olato)-κO^1^-κO^2^)(η^6^-p-cymenyl)ruthenium(II)] **(5)**	IC_50_ = 1.2 ± 0.2 µM (CH1/PA-1, after 96 h) IC_50_ = 0.094 ± 0.031 µM (SW480, after 96 h) IC_50_ = >50 µM (A549, after 96 h)	Cseh et al. (2022) [138]
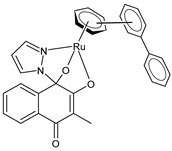	[3-Methyl-4-oxo-(1*H*-κN^2^-pyrazol-1-yl)-1,4-dihydronaphtalene-1,2-bis(olato)-κO^1^-κO^2^)(η^6^-biphenyl)ruthenium(II)] **(6)**	IC_50_ = 1.2 ± 0.2 µM (CH1/PA-1, after 96 h) IC_50_ = 0.072 ± 0.019 µM (SW480, after 96 h) IC_50_ = 30 ± 3 µM (A549, after 96 h)	Cseh et al. (2022) [138]
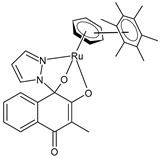	[3-Methyl-4-oxo-(1*H*-κN^2^-pyrazol-1-yl)-1,4-dihydronaphtalene-1,2-bis(olato)-κO^1^-κO^2^)(η^6^-hexamethylbenzene)ruthenium(II)] **(7)**	IC_50_ = 3.4 ± 0.6 µM (CH1/PA-1, after 96 h) IC_50_ = 0.27 ± 0.06 µM (SW480, after 96 h) IC_50_ = 35 ± 4 µM (A549, after 96 h)	Cseh et al. (2022) [138]
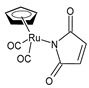	η^5^-cyclopentadienyl)Ru (CO)_2_(*η*^1^-*N*-maleimidato **(8)**	IC_50_ = 5.62 µM (HL-60)	Juszczak et al. (2022) [140]
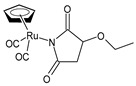	(*η*^5^-cyclopentadienyl)Ru(CO)_2_-*N*-ethoxysuccinimidato **(9)**	IC_50_ > 250 µM (HL-60)	Juszczak et al. (2022) [140]
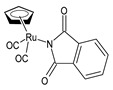	*η*^5^-cyclopentadienyl)Ru(CO)_2_-*N*-phthalimidato **(10)**	IC_50_ > 250 µM (HL-60)	Juszczak et al. (2022) [140]
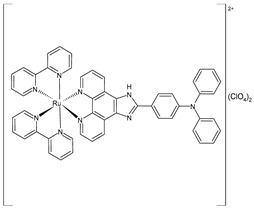	[Ru(2,2′-bipyridine)_2_(IPP)](ClO_4_)_2_ **(11)**	IC_50_ = 15.1 ± 0.2 µM (B16) IC_50_ = 19.7 ± 1.4 µM (HepG2) IC_50_ = 16.9 ± 0.7 µM (A549)	Liang et al. (2022) [141]
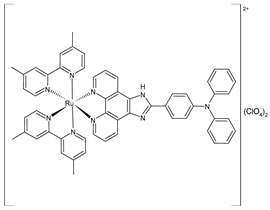	[Ru(4,4′-dimethyl-2,2′-bipyridine)_2_(IPP)](ClO_4_)_2_ **(12)**	IC_50_ = 14.3 ± 0.1 µM (B16) IC_50_ = 19.1 ± 1.7 µM (HepG2) IC_50_ = 13.0 ± 0.5 µM (A549)	Liang et al. (2022) [141]
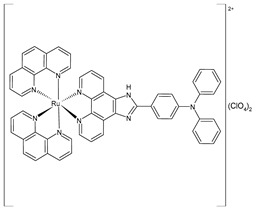	[Ru(1,10-phenanthroline)_2_(IPP)](ClO_4_)_2_ **(13)**	IC_50_ = 26.0 ± 2.1 µM (B16) IC_50_ = 36.8 ± 1.7 µM (HepG2) IC_50_ = 32.3 ± 0.4 µM (A549)	Liang et al. (2022) [141]
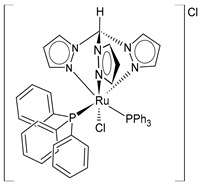	[RuCl(κ^3^-tris(1-pyrazolyl)methane)(PPh_3_)_2_]Cl **(14)**	IC_50_ = 2.4 ± 0.6 µM (MCF-7) IC_50_ = 4.0 ± 0.4 µM (HeLa) IC_50_ = 2.6 ± 0.4 µM (518A2) IC_50_ = 1.5 ± 0.1 µM (HCT-116) IC_50_ = 2.2 ± 0.2 µM (RD)	Cervinka et al. (2022) [142]
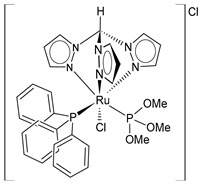	[RuCl(κ^3^-tris(1-pyrazolyl)methane) (PPh_3_){P(OMe)_3_}]Cl **(15)**	IC_50_ = 6 ± 1 µM (MCF-7) IC_50_ = 10 ± 2 µM (HeLa) IC_50_ = 6.8 ± 0.8 µM (518A2) IC_50_ = 6.7 ± 0.4 µM (HCT-116) IC_50_ = 6 ± 1 µM (RD)	Cervinka et al. (2022) [142]
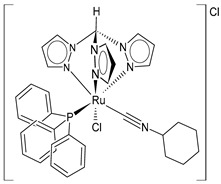	[RuCl(κ^3^-tris(1-pyrazolyl)methane)(PPh_3_)(CNCy)]Cl **(16)**	IC_50_ = 10 ± 2 µM (MCF-7) IC_50_ = 15 ± 1 µM (HeLa) IC_50_ = 10 ± 2 µM (518A2) IC_50_ = 8 ± 2 µM (HCT-116) IC_50_ = 6.6 ± 0.7 µM (RD)	Cervinka et al. (2022) [142]
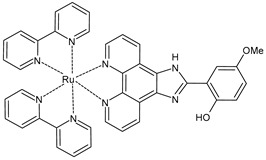	[Ru(bpy)_2_L](ClO_4_)_2_ **(17)**	IC_50_ = 99.80 ± 1.9 (HeLa) µM (after 24 h)	Priya et al. (2023) [143]
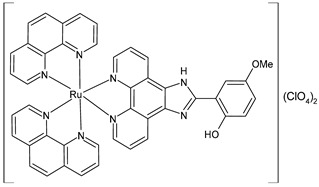	[Ru(phenyl)_2_L](ClO_4_)_2_ **(18)**	IC_50_ = 24.5 ± 1.45 µM (HeLa) (after 24 h)	Priya et al. (2023) [143]
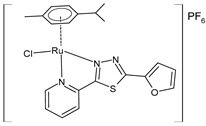	[Ru(η^6^-pcym)(L1)Cl]PF_6_ **(19)**	IC_50_ = 8.69 ± 1.75 µM (A2780, 48 h) IC_50_ = 12.48 ± 4.83 µM (A2780cis) (after 48 h)	Křikavová et al. (2023) [144]
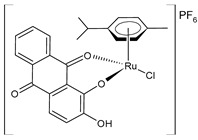	[Ru(L)Cl(η^6^-*p*-cymene)] **(20)**	IC_50_ = 42.2 ± 3.6 µM (MDA-MB-231) IC_50_ = 32.8 ± 1.2 µM (MCF-7) IC_50_ > 100 µM (A549)	de Araujo-Neto et al. (2023) [145]
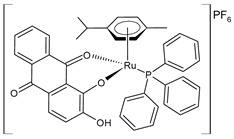	[Ru(L)(η^6^-*p*-cymene)(PPh_3_)]PF_6_ **(21)**	IC_50_ = 6.5 ± 0.1 µM (MDA-MB-231) IC_50_ = 9.0 ± 0.1 µM (MCF-7) IC_50_ = 17.8 ± 0.8 µM (A549)	de Araujo-Neto et al. (2023) [145]
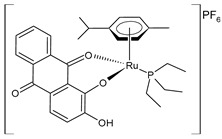	[Ru(L)(η^6^-*p*-cymene)(PEt_3_)]PF_6_ **(22)**	IC_50_ = 45.4 ± 1.4 µM (MDA-MB-231) IC_50_ > 100 µM (MCF-7) IC_50_ = 52.6 ± 1.2 µM (A549)	de Araujo-Neto et al. (2023) [145]
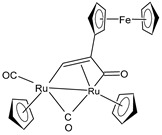	[Ru_2_Cp_2_(CO)(μ-CO){μ-η^1^:η^3^-CH=C(Fc)C(=O)}] **(23)**	IC_50_ > 100 µM (A549) IC_50_ > 100 µM (SW480) IC_50_ = 4.1 ± 0.9 µM (A2780) IC_50_ = 4.1 ± 0.9 µM (A2780cis)	Bresciani et al. (2023) [146]
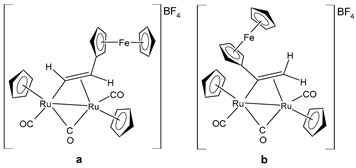	[Ru_2_Cp_2_(CO)_2_(μ-CO){μ-η^1^:η^2^-CH=CH(Fc)}]BF_4_ **(24a)** [Ru_2_Cp_2_(CO)_2_(μ-CO){μ-η^1^:η^2^-C(Fc)CH_2_}]BF_4_ **(24b)**	IC_50_ = 41 ± 5 µM (A549) IC_50_ = 38 ± 2 µM (SW480) IC_50_ = 8 ± 4 µM (A2780) IC_50_ = 11.0 ± 0.2 µM (A2780cis)	Bresciani et al. (2023) [146]
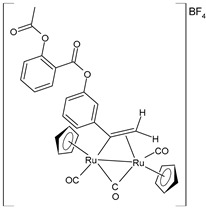	[Ru_2_Cp_2_(CO)_2_(μ-CO){μ-η1:η^2^-C(3-C_6_H_4_-Asp)=CH_2_}]BF_4_ **(25)**	IC_50_ = 19 ± 3 µM (A549) IC_50_ = 22 ± 2 µM (SW480) IC_50_ = 7.9 ± 1.3 µM (A2780) IC_50_ = 9.0 ± 1.3 µM (A2780cis)	Bresciani et al. (2023) [146]
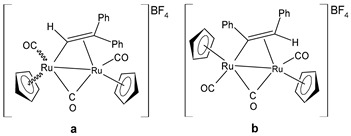	[Ru_2_Cp_2_(CO)_2_(μ-CO){μ-η^1^:η^2^-C(H)CPh_2_}]BF_4_ **(26a)** [Ru_2_Cp_2_(CO)_2_(μ-CO){μ-η^1^:η^2^-C(Ph)CH(Ph)}]BF_4_ **(26b)**	IC_50_ = 34 ± 2 µM (A549) IC_50_ = 34 ± 2 µM (SW480) IC_50_ = 8.5 ± 6 µM (A2780) IC_50_ = 10.6 ± 0.8 µM (A2780cis)	Bresciani et al. (2023) [146]
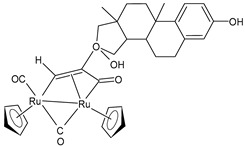	[Ru_2_Cp_2_(CO)2{µ-η^1^:η^3^-CH=C(17α-estradiol)C(=O)}] **(27)**	IC_50_ = 6.2 ± 1.2 µM (A2780) IC_50_ = 7.3 ± 2.4 µM (A2780cisR) IC_50_ = 19.0 ± 4.5 µM (MCF-7) IC_50_ = 24.0 ± 3.8 µM (HOS) IC_50_ > 50 µM (A549) IC_50_ > 50 µM (PANC-1) IC_50_ >50 µM (Caco-2) IC_50_ = 36.0 ± 4.1 µM (PC-3) IC_50_ = 5.5 ± 0.9 µM (HeLa)	Bresciani (2023) [147]
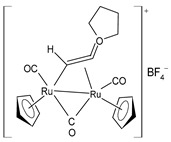	[Ru_2_Cp_2_(CO)_3_{µ-η^1^:η^3^-CH=C= (cyclopentylidene)}]BF_4_ **(28)**	IC_50_ = 4.2 ± 0.9 µM (A2780) IC_50_ = 6.4 ± 1.9 µM (A2780cisR) IC_50_ = 16.2 ± 1.7 µM (MCF-7) IC_50_ = 14.6 ± 0.5 µM (HOS) IC_50_ = 25.3 ± 1.9 µM (A549) IC_50_ = 28.4 ± 3.9 µM (PANC-1) IC_50_ > 50 µM (Caco-2) IC_50_ = 22.2 ± 2.4 µM (PC-3) IC_50_ = 17.5 ± 2.9 µM (HeLa)	Bresciani (2023) [147]
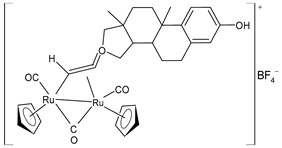	[Ru_2_Cp_2_(CO)_3_{µ-η^1^:η^2^-CH=C= (estradiolylidene)}]BF_4_ **(29)**	IC_50_ = 3.4 ± 0.6 µM (A2780) IC_50_ = 4.6 ± 1.3 µM (A2780cisR) IC_50_ = 11.6 ± 1.5 µM (MCF-7) IC_50_ = 12.6 ± 0.5 µM (HOS) IC_50_ = 16.1 ± 1.3 µM (A549) IC_50_ = 19.8 ± 2.3 µM (PANC-1) IC_50_ = 36.0 ± 2.7 µM (Caco-2) IC_50_ = 42.8 ± 0.8 µM (PC-3) IC_50_ = 5.5 ± 0.9 µM (HeLa)	Bresciani (2023) [147]
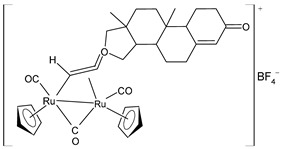	[Ru_2_Cp_2_(CO)_3_{µ-η^1^:η^2^-CH=C= (testosteronylidene)}]BF_4_ **(30)**	IC_50_ = 6.3 ± 1.3 µM (A2780) IC_50_ = 11.7 ± 2.4 µM (A2780cisR) IC_50_ = 22.0 ± 4.0 µM (MCF-7) IC_50_ = 17.7 ± 2.8 µM (HOS) IC_50_ = 20.7 ± 1.4 µM (A549) IC_50_ = 30.0 ± 0.6 µM (PANC-1) IC_50_ = 42.8 ± 0.8 µM (Caco-2) IC_50_ = 19.6 ± 3.7 µM (PC-3) IC_50_ = 16.3 ± 1.3 µM (HeLa)	Bresciani (2023) [147]
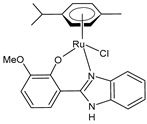	[Ru(η^6^-p-cym)(L)Cl] **(31)**	IC_50_ = 11.84 ± 0.42 µM (HeLa) IC_50_ = 25.67 ± 0.56 µM (MCF-7)	Nayek et al. (2023) [148]
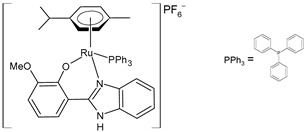	[Ru(η^6^-p-cym)(L)PPh_3_]PF_6_ **(32)**	IC_50_ = 7.29 ± 0.38 µM (HeLa) IC_50_ = 19.97 ± 0.39 µM (MCF-7)	Nayek et al. (2023) [148]
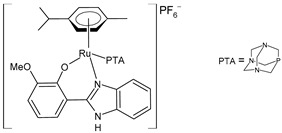	[Ru(η^6^-p-cym)(L)(PTA)]PF_6_ **(33)**	IC_50_ = 13.25 ± 0.35 µM (HeLa) IC_50_ = 28.70 ± 0.48 µM (MCF-7)	Nayek et al. (2023) [148]
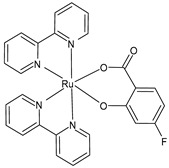	[Ru(bipy)2(4-F-Sal)] **(34)**	IC_50_ = 5.76 × 10^−6^ M; 4.75 × 10^−6^ M (MCF-7, after 24 h and 48 h, respectively) IC_50_ = > 10 × 10^−6^ M (U-118MG, after 24 h and 48 h)	Schoeller et al. (2023) [149]
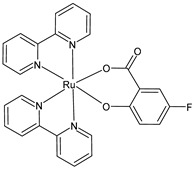	[Ru(bipy)_2_(5-F-Sal)] **(35)**	IC_50_ = < 2 × 10^−6^ M (MCF-7, after 24 h and 48 h) IC_50_ = 3.56 × 10^−6^ M; 4.72 × 10^−6^ M (U-118MG, after 24 h and 48 h, respectively)	Schoeller et al. (2023) [149]
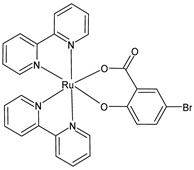	[Ru(bipy)_2_(5-Br-Sal)] **(36)**	IC_50_ = 4.23 × 10^−6^ M; 4.92 × 10^−6^ M (MCF-7, after 24 h and 48 h, respectively) IC_50_ = 5.35 × 10^−6^ M; 3.95 × 10^−6^ M (U-118MG, after 24 h and 48 h, respectively)	Schoeller et al. (2023) [149]
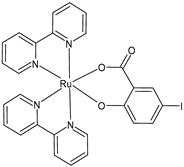	[Ru(bipy)_2_(5-I-Sal)] **(37)**	IC_50_ < 2 × 10^−6^ M (MCF-7, after 24 h and 48 h) IC_50_ = 4.08 × 10^−6^ M; 2.65 × 10^−6^ M (U-118MG, after 24 h and 48 h, respectively)	Schoeller et al. (2023) [149]
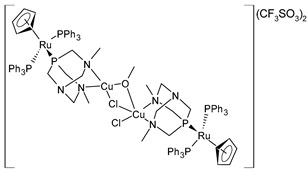	[{RuCp(PPh_3_)_2_-μ-dmoPTA-1κP:2κ^2^-*N*,*N*′-CuCl}_2_-μ-Cl-μ-OCH_3_](CF_3_SO_3_)_2_·(CH_3_OH)_4_ **(38)**	GI_50_ = 28 ± 3.3 nM (A549, after 48 h) GI_50_ = 32 ± 0.2 nM (HBL-100, after 48 h) GI_50_ = 21 ± 1.7 nM (HeLa, after 48 h) GI_50_ = 27 ± 13 nM (SW1573, after 48 h) GI_50_ = 20 ± 7.8 nM (T-47D, after 48 h) GI_50_ = 21 ± 9.2 nM (WiDr, after 48 h)	Alguacil et al. (2023) [150]
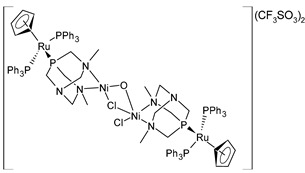	[{RuCp(PPh_3_)_2_-μ-dmoPTA-1κP:2κ^2^-*N,N*′-NiCl}_2_-μ-Cl-μ-OH](CF_3_SO_3_)_2_ **(39)**	GI_50_ = 34 ± 8.4 nM (A549, after 48 h) GI_50_ = 31 ± 11 nM (HBL-100, after 48 h) GI_50_ = 28 ± 2.5 nM (HeLa, after 48 h) GI_50_ = 41 ± 6.8 nM (SW1573, after 48 h) GI_50_ = 23 ± 1.6 nM (T-47D, after 48 h) GI_50_ = 34 ± 8.7 nM (WiDr, after 48 h)	Alguacil et al. (2023) [150]
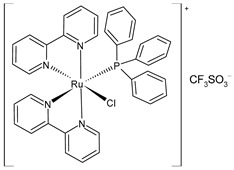	cis-[Ru(bpy)_2_(PPh_3_)Cl] CF_3_SO_3_ **(40)**	IC_50_ = 73.31 ± 0.10 µM and 1.16 ± 0.10 µM (HL-60, after 24 h and 72 h, respectively) IC_50_ = 3.45 ± 0.99 µM (A549, after 24 h) IC_50_ = 1.62 ± 0.33 µM (DU145, after 24 h). IC_50_ = 13.58 ± 2.11 µM (HeLa, after 24 h)	Mitchell et al. (2023) [151]
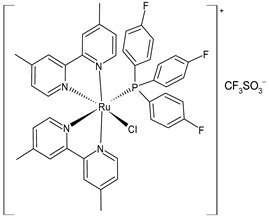	cis-[Ru4.4′-Me_2_bpy)_2_{P(C_6_H_4_F)_3_}Cl] CF_3_SO_3_ **(41)**	IC_50_ = 2.74 ± 0.56 µM and 0.98 ± 0.24 µM (HL-60, after 24 h and 72 h) IC_50_ = 2.78 ± 0.34 µM (A549, after 24 h) IC_50_ = 1.42 ± 0.20 µM (DU145, after 24 h). IC_50_ = 5.67 ± 2.19 µM (HeLa, after 24 h)	Mitchell et al. (2023) [151]
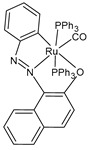	[Ru(L^1^)(CO)(PPh_3_)_2_] **(42)**	IC_50_ = 5.1 ± 1.2 µM (MCF-7) IC_50_ = 36.2 ± 1.5 µM (A549) IC_50_ = 65.3 ± 1.2 µM (MDA-MB-231) IC_50_ = 42.1 ± 3.1 µM (AGS)	Das et al. (2023) [152]
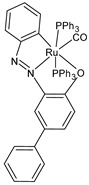	[Ru(L^2^)(CO)(PPh_3_)_2_] **(43)**	IC_50_ = 6.3 ± 3.1 µM (MCF-7) IC_50_ = 21.3 ± 3.2 µM (A549) IC_50_ = 53.2 ± 1.3 µM (MDA-MB-231) IC_50_ = 51.1 ± 1.4 µM (AGS)	Das et al. (2023) [152]
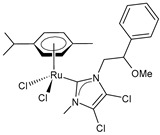	1-(2-methoxy-2-phenylethyl)-3-methyl) (4,5-dichloroimidazol-2-ylidene) (*p*-cymene) ruthenium(II) chloride **(44)**	IC_50_ = 24.14 ± 0.07 µM (MDA-MB-231) IC_50_ = 26.05 ± 0.9 µM (MCF-7) IC_50_ = 48.43 ± 0.8 µM (SH-SY5Y)	Ceramella et al. (2023) [153]
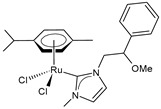	1-(2-methoxy-2-phenylethyl)-3-methyl-imidazol-2-ylidene) (*p*-cymene) ruthenium(II) chloride **(45)**	IC_50_ = 40.57 ± 1.1 µM (MDA-MB-231) IC_50_ = 54.75 ± 1.1 µM (MCF-7) IC_50_ = 66.86 ± 0.8 µM (SH-SY5Y)	Ceramella et al. (2023) [153]
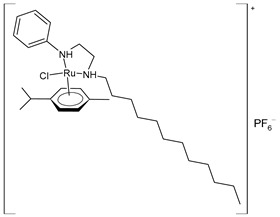	**(46)**	Cell viability reduction = ~50% at 10 nM (HepG2)	Kavukcu et al. (2023) [154]
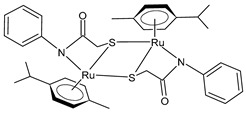	**(47)**	Cell viability reduction = ~50% at 10 nM (HepG2)	Kavukcu et al. (2023) [154]
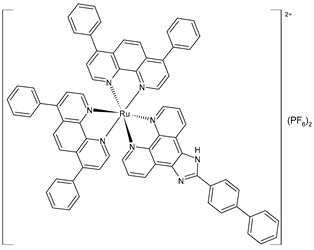 liposome	[Ru(4,7-diphenyl-1,10-phenanthroline)_2_(2-(1,1′-biphenyl-4-yl)-1*H*-imidazo [4,5-f][1,10]phenanthroline)](PF_6_)_2_ **(48)lipo**	IC_50_ = 9.3 ± 0.3 µM (A549) IC_50_ = 17.4 ± 0.3 µM (HepG2) IC_50_ = 3.4 ± 0.1 µM (SGC-7901) IC_50_ = 14.8 ± 0.4 µM (HeLa) IC_50_ = 5.9 ± 0.2 µM (Bel-7402) IC_50_ = 7.2 ± 0.2 µM (B16)	Chen et al. (2023) [155]
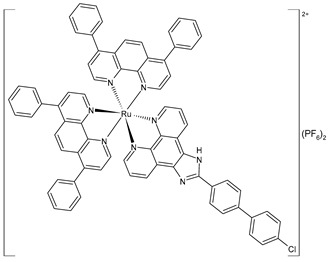 liposome	[Ru(4,7-diphenyl-1,10-phenanthroline)_2_(2-(4′-chloro-1,1′-biphenyl-4-yl)-1*H*-imidazo [4,5-f][1,10]phenanthroline)](PF_6_)_2_ **(49)lipo**	IC_50_ = 7.7 ± 0.2 µM (A549) IC_50_ = 15.0 ± 0.2 µM (HepG2) IC_50_ = 3.5 ± 0.1 µM (SGC-7901) IC_50_ = 14.7 ± 0.6 µM (HeLa) IC_50_ = 5.8 ± 0.1 µM (Bel-7402) IC_50_ = 5.1 ± 0.1 µM (B16)	Chen et al. (2023) [155]

### 4.2. Preclinical In Vitro Studies and In Silico Studies on Ru(II) Complexes as Promising Dual-Active Agents against Cancer and Viruses

Santi et al. (2021) [156] (Table 2) studied the activity of Ru(II) η^6^–arene compounds using 3D models of head and neck squamous carcinoma cells (HNSCCs) with or without human papilloma virus (HPV) infection compared with RAPTA-C. Human squamous cell carcinomas SCC-25 (HPV-negative, HPV−) and UPCI-SCC-154 (HPV-positive, HPV+) were used (IC_50_ values for RAPTA-C were >400 µM for both). Complex **50** showed a promising cytotoxic effect on all the tested cell lines in both 2D and 3D cell cultures. Importantly, this complex showed higher activity regarding the HPV− carcinoma, which is typically more aggressive, usually has a poorer prognosis, and has a higher risk of recurrence/metastasis in comparison to the HPV+ one. Recently, cisplatin and **50**—both as free molecules—have been loaded into hybrid nanoarchitectures (NAs), thereby showing a supraadditive action in both 2D and 3D models of HPV− HNSCC, thereby suggesting a possible reduction in the dose of cisplatin administered to patients, which, in turn, may lead to a reduction in side effects and result in a better prognosis [157]. The combined effect was also evaluated on the chorioallantoic membranes (CAMs), which are in vivo alternative models for the reliable evaluation of innovative approaches for cancer detection and treatment. This study evidenced the biosafety, the NA activity, and the lack of Ru(II) bioaccumulation in major organs.

Janković et al. (2022) [135] recently reported a finalized study regarding the discovery of dual-active agents acting as anticancer and antiviral agents, which was based on the hybridization concept of “one drug curing two diseases” potentially being a successful tactic in healing patients who have cancer and the virus SARS-CoV-2 at the same time. The cytotoxicity effects of the half-sandwich Ru complexes containing Biginelli hybrids (**51**–**55**) were evaluated against the human cancer cell lines of cervical adenocarcinoma (HeLa), lung carcinoma (A549), colon adenocarcinoma (LS174), malignant melanoma (A375), and chronic myelogenous leukemia (K562), as well as against one normal human cell line, lung fibroblast (MRC-5) cells, via an MTT assay. Cisplatin was used as the reference drug (IC_50_ = 2.36 ± 0.28 µM; 17.93 ± 0.44 µM; 20.8 ± 0.44 µM; 2.56 ± 0.42 µM; and 5.56 ± 0.23 µM, respectively, against cancerous cells). The anticancer activity effects were examined against a human umbilical vein cell line, EA.hy926, using an MTT test. The complexes that showed the highest cytotoxic activities, **52** and **53**, were then chosen to analyze their effects on the distribution of HeLa cells in the cell-cycle phases using flow cytometry analysis. The results suggested that the proportion of cells in the G2/M phase decreased following the increase in the sub-G1 phase in all treatments, thus confirming that cells treated with **52** and **53** were induced to undergo apoptotic death. In silico studies using AutoDock showed the significant inhibitory potency of the complexes against SARS-CoV-2 M^pro^ (PDB ID: **6LU7**). Docking studies revealed that the M^pro^-55 protein–ligand complex showed the lowest values of free energy of binding (ΔG_bind_) and *K*_i_ (−7.34 kcal/mol and 4.18 μM, respectively), which are comparable to those of cinanserin (−7.81 kcal/mol and 1.88 μM, respectively) and hydroxychloroquine (−7.00 kcal/mol and 7.43 μM, respectively). Complex **52** was suggested to become a possible candidate for dual therapy (anticancer–antiviral) in the future.

Wang et al. (2022) [158] reported on the study of four polypyridyl Ru(II) complexes (**56**–**59**) as bifunctional TAR RNA binders and HIV-1 reverse transcriptase (RT) inhibitors. Molecular recognition of the hydrogen bonds further stabilized the Ru(II)–RNA-bound system through electrostatic attraction, which efficiently inhibited the Moloney murine leukemia virus (M-MuLV) and HIV-1 RTs. The former was evaluated by determining the IC_50_ value, that is, the inhibitory concentration that prevented 50% of the poly(A) RNA to be reverse-transcribed to poly(dT) cDNA by the M-MuLV RTs, whereas for the latter, the EC_50_ value (that is, the effective concentration required to cause 50% inhibition activity toward the HIV-1 RTs) was determined in comparison to etravirine (EC_50_ = 0.0177 ± 0.0014 µM). The polypyridyl Ru(II) complexes also have physical and chemical advantages, such as high chemical stability and photostability, sensitive spectroscopic responses to HIV TAR RNA, and low toxicity to normal cells. Cytotoxicity assays for normal human liver (HL-7702) cells were also performed in comparison to etravirine. All of the Ru(II) complexes exhibited low cytotoxicity activities, with their CC_50_ values (defined as the cytotoxic concentration of the compound that reduces the viability of the HL-7702 cells by 50%) being almost an order of magnitude lower than that of etravirine (CC_50_ = 21.7 ± 1.6 µM).

An interesting study was recently carried out by Li et al. (2023) [159], who suggested a new anti-influenza drug (**60**) prepared using Ru and selenium (Se) acting against the influenza A (H1N1) virus, which is responsible for an acute respiratory infectious disease that causes massive morbidity and mortality worldwide. The RuSe compound significantly inhibited MDCK cell apoptosis induced by H1N1; it inhibited the replication and proliferation of the influenza virus by inhibiting nucleoprotein (NP) nuclear export. In vivo experiments in mice showed that the RuSe compound inhibited H1N1-mediated apoptosis by regulating the proteins associated with the apoptotic pathway. In vitro, RuSe exhibited a certain direct antiviral action, thereby demonstrating certain inhibitory effects on the virulence, nucleic acid replication, NA activity, and influenza protein expression of H1N1. As an anti-influenza drug, RuSe played an antiviral role and also acted as a drug carrier to deliver selenium to the organism, regulate the selenium proteins GPx1 and TrxR1 in vivo, and play an antioxidant role in inhibiting ROS-mediated apoptosis. The antiviral activity of **60** was evaluated by measuring the virulence of the progeny viruses of the H1N1 group and the H1N1+**60** group (TCID_50_, a median-tissue-culture infective dose). The virulence of the progeny virus in the H1N1 group was 3.09·10^5^/0.1 mL, whereas that in the H1N1+**60** group was 1.04·10^2^/0.1 mL. The virulence of the progeny virus of the treatment group was significantly reduced. At the same time, the determination of the nucleoprotein (NP) of the influenza virus showed that the relative NP expression of the H1N1+**60** group was 32.6% that of the H1N1 group. Moreover, the relative neuraminidase activity of the H1N1+**60** group was 66.3%.

## 5. Conclusions

Ru complexes are currently objects of considerable attention in therapy, especially as antitumor agents with selective antimetastatic properties and low systemic toxicity. NAMI-A and BOLD-100 are structurally related Ru(III) coordination compounds that have attracted a lot of attention in the medicinal inorganic chemical scientific community for their anticancer activities. Ru(II) complexes have the potential to provide a safer, more-effective, and less-expensive alternative to traditional platinum-based chemotherapy for biomedical applications. Recent studies have been focused towards the synthesis of new analogues of RAED-C and RAPTA-C, which are two Ru(II) complexes that have shown excellent antitumoral activities in both in vitro and in vivo studies. The most interesting results were obtained with complexes bearing the *p*-cymene moiety, including 1,3,5-triaza-7-phosphaadamantane and triphenylphosphine, as well as with polypyridyl and NHC derivatives. Interesting recent studies have also been focused on complexes bearing more than one transition metal type, such as Cu, Ni, and Se, thereby obtaining high activity effects, also in the nanomolar range, against different cell lines. The challenge is now represented by the discovery of new dual-active drugs that act as anticancer and antiviral agents. Although there are a lot of studies on antitumoral activity, very few studies have been carried out regarding antiviral activity. Interestingly, BOLD-100 has demonstrated activity against SARS-CoV-2, HIV-1, and human adenovirus type 5. Thus, new studies are needed in this direction. The search for effective alternatives to existing transition metal complexes used in therapy or under clinical trials is still a great challenge for scientists. The major goal is represented by the need to overcome the most common limitations, such as the onset of resistance phenomena and severe side effects. The employment of Ru complexes may offer a valid alternative to the most-used platinum drugs because of their lower toxicity, synergistic features, and the ability to overcome drug resistance. The discussed different chemical properties and the versatility of the obtained complexes represent the winning points for the future development and new applications of these complexes as part of a valid therapeutic arsenal.

The demonstrated effectiveness of Ru(II) complexes and, generally, the coordination of Ru(II) with different ligands are vital for their activity and selectivity effects. Thus, future studies should focus on investigating the structure–activity relationships (SARs) in order to establish the role of different functional groups interacting with the ligands in modulating the activity effects. Furthermore, the diffusion across the cell membrane and the possibility to target organelles, such as mitochondria, or important biomolecules, such as DNA and proteins, should be studied in association with the charge or lipophilicity of the considered complexes in order to design and synthetize more nontoxic and selective drugs. Next, it should also be highlighted that a growing trend is being directed toward the design of hybrid complexes, made of Ru complex moieties combined with natural biomolecules or fluorescent probes, whose applications would have a high potential in different research and clinical fields. Finally, one of the major hindrances regarding the development of Ru(II) complexes and their application in clinics is that their mechanisms of action are still poorly investigated and understood, from which the need for in-depth studies is highly desirable.

In conclusion, based on an in-depth study of the analyzed papers, it can be deduced that Ru(II) complexes could represent very promising compounds, with dual activity as anticancer and antiviral agents, as well as low toxicity. Finally, studies regarding Ru complexes with liposomes and NAs may shed new light in this scenario.

## Figures and Tables

**Table 2:** In vitro studies and in silico studies on Ru(II) complexes as anticancer and antiviral agents.

Structure	Compound	Cytotoxicity Studies	Antiviral Studies	Ref.
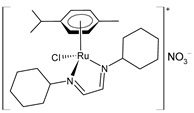	[RuCl{κ^2^*N*-(HC=N-cyclohexyl)_2_}(η^6^-*p*-cymene)]NO_3_ **(50)**	IC_50_ = 78.5 µM (SCC-25, after 72 h) IC_50_ = 91.8 µM (UPCI-SCC-154, after 72 h)		Santi et al. (2021) [156]
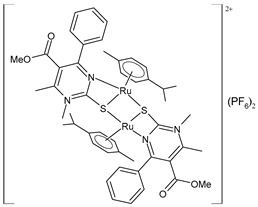	[(*p*-cymene)Ru(4a)]_2_ (PF_6_)_2_ **(51)**	IC_50_ = 34.70 ± 1.23 µM (HeLa) IC_50_ = 61.99 ± 0.36 µM (A549) IC_50_ = 67.43 ± 1.24 µM (LS174) IC_50_ = 14.14 ± 1.11 µM (A375) IC_50_ = 11.44 ± 1.19 µM (K652) IC_50_ = 59.96 ± 11.50 μM (EA.hy926)	ΔG_bind_ = −6.40 kcal/mol *K*_i_ = 20.25 μM	Janković et al. (2022) [135]
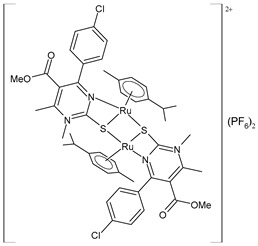	[(*p*-cymene)Ru(4b)]_2_ (PF_6_)_2_ **(52)**	IC_50_ = 16.39 ± 0.43 µM (HeLa) IC_50_ = 24.87 ± 1.14 µM (A549) IC_50_ = 32.78 ± 3.38 µM (LS174) IC_50_ = 14.00 ± 0.10 µM (A375) IC_50_ = 11.45 ± 0.15 µM (K652) IC_50_ = 35.24 ± 1.08 μM (EA.hy926)	ΔG_bind_ = −6.24 kcal/mol *K*_i_ = 26.84 μM	Janković et al. (2022) [135]
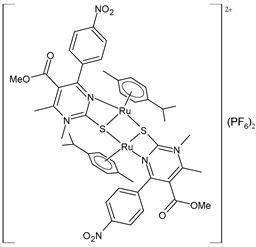	[(*p*-cymene)Ru(4c)]_2_ (PF_6_)_2_ **(53)**	IC_50_ = 17.89 ± 0.7 µM (HeLa) IC_50_ = 33.85 ± 2.74 µM (A549) IC_50_ = 34.00 ± 1.39 µM (LS174) IC_50_ = 13.94 ± 0.25 µM (A375) IC_50_ = 8.63 ± 0.24 µM (K652) IC_50_ = 33.85 ± 1.68 μM (EA.hy926)	ΔG_bind_ = −5.53 kcal/mol *K*_i_ = 88.62 μM	Janković et al. (2022) [135]
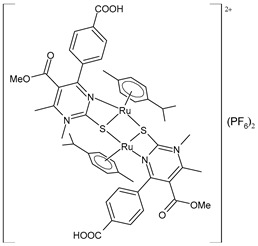	[(*p*-cymene)Ru(4d)]_2_ (PF_6_)_2_ **(54)**	IC_50_ = 69.66 ± 4.33 µM (HeLa) IC_50_ = not active (A549) IC_50_ = 81.79 ± 4.28 µM (LS174) IC_50_ = 199.53 ± 0.67 µM (A375) IC_50_ = 198.09 ± 1.58 µM (K652) IC_50_ = not active (EA.hy926)	ΔG_bind_ = −5.32 kcal/mol *K*_i_ = 124.98 μM	Janković et al. (2022) [135]
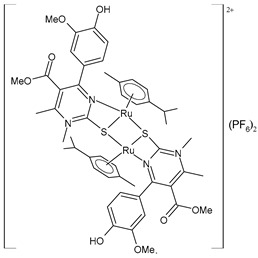	[(*p*-cymene)Ru(4e)]_2_ (PF_6_)_2_ **(55)**	IC_50_ = 78.28 ± 3.26 µM (HeLa) IC_50_ = not active (A549) IC_50_ = 97.77 ± 1.43 µM (LS174) IC_50_ = 116.66 ± 5.72 µM (A375) IC_50_ = 130-48 ± 3.13 µM (K652) IC_50_ = not active (EA.hy926)	ΔG_bind_ = −7.34 kcal/mol *K*_i_ = 4.18 μM	Janković et al. (2022) [135]
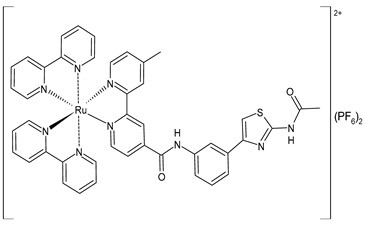	[Ru(bpy)_2_(L1)] (PF_6_)_2_ **(56)**	CC_50_ = 226 ± 12 µM (HL-7702 normal cells)	IC_50_ = 1.85 ± 0.09 µM (M-MuLV RT) EC_50_ = 0.168 ± 0.009 µM (HIV-RT)	Wang et al. (2022) [158]
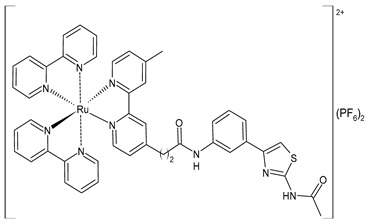	[Ru(bpy)_2_(L_2_)] (PF_6_)_2_ **(57)**	CC_50_ = 247 ± 11 µM (HL-7702 normal cells)	IC_50_ = 3.62 ± 0.10 µM (M-MuLV RT) EC_50_ = 0.357 ± 0.023 µM (HIV-RT)	Wang et al. (2022) [158]
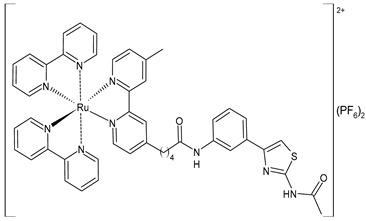	[Ru(bpy)_2_(L_3_)] (PF_6_)_2_ **(58)**	CC_50_ = 239 ± 16 µM (HL-7702 normal cells)	IC_50_ = 4.74 ± 0.11 µM (M-MuLV RT) EC_50_ = 0.446 ± 0.032 µM (HIV-RT)	Wang et al. (2022) [158]
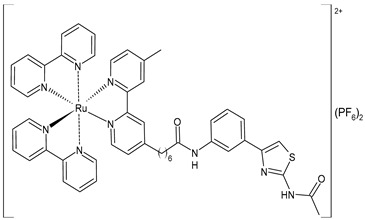	[Ru(bpy)_2_(L_4_)] (PF_6_)_2_ **(59)**	CC_50_ = 231 ± 18 µM (HL-7702 normal cells)	IC_50_ = 5.49 ± 0.26 µM (M-MuLV RT) EC_50_ = 0.522 ± 0.032 µM (HIV-RT)	Wang et al. (2022) [158]
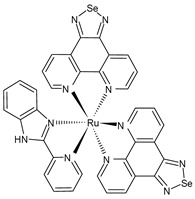	Ru(biim) (PhenSe)_2_ **(60)**		TCID_50_ = 1.04·10^2^/0.1 mL (H1N1+ RuSe group)	Li et al. (2023) [159]

## Data Availability

Data sharing is not applicable.

## Abbreviations and Cancer Cells Mentioned in the Text

518A2	melanoma cell lines
A375	malignant melanoma cell lines
A2780	ovarian cancer cell lines
A2780cis	ovarian cancer cell lines
A2780cisR	cisplatin-resistant human ovarian carcinoma
A549	lung cancer cells
AGS	gastric adenocarcinoma cell line
B16	mouse melanoma cells
BALB/3T3	non-tumoral cells
BEL-7402	hepatocellular carcinoma
Caco-2	human colorectal adenocarcinoma
CCD-18Co	colon healthy cell lines
CCD-1072Sk	foreskin fibroblasts healthy cell lines
CH1/PA-1	teratocarcinoma cells
DU145	prostate adenocarcinoma cells
HCT-116	human colon cancer cells
HeLa	human cervix adenocarcinoma cancer cells
HBL-100	breast cancer cells
HepG2	human liver cancer cells
HEK293	human embryonic kidney nontumoral cell lines
HIV-1	type 1 human immunodeficiency virus
HNSCCs	head and neck squamous carcinoma cells
IC50	half-maximal (50%) inhibitory concentration
HOS	human osteosarcoma
HPV	human papillomavirus
K562	chronic myelogenous leukemia cells
LS174	colon adenocarcinoma cells
MCF-7	breast cancer cells
MCF-10A	nontumor breast cell lines
MDA-MB-231	triple negative breast cancer cells
MTT	3-(4,5-dimethylthiazol-2-yl)-2,5-diphenyltetrazolium bromide
MRC-5	non-tumor lung cell lines
MRC5pd30	normal human fibroblasts
NAs	nano-architectures
PANC-1	human pancreatic carcinoma cells
PC-3	human prostate carcinoma cells
PLpro	papain-like protease
RD	rhabdomyo-sarcoma cells
SCC-25	human squamous cell carcinoma (HPV-negative)
SW480	colon adenocarcinoma cell lines
SW1573	lung cancer cells
ROS	reactive oxygen species
SGC-7901	gastric adenocarcinoma
SiHa	human cervical cancer cells
TCID50	median tissue culture infective dose
U-118MG	glioma cell lines
UPCI-SCC-154	human squamous cell carcinoma (HPV-positive)
WiDr	colon cancer cells
